# The effects on type 2 diabetes mellitus mouse femoral bone achieved by anti-osteoporosis exercise interventions

**DOI:** 10.3389/fendo.2022.914872

**Published:** 2022-11-18

**Authors:** Miao Zhang, Yuexuan Li, Lifei Liu, Mei Huang, Miao Wang, Jun Zou

**Affiliations:** ^1^ School of Exercise and Health, Shanghai University of Sport, Shanghai, China; ^2^ Department of Rehabilitation, The People’s Hospital of Liaoning Province, Shenyang, China

**Keywords:** T2DM, exercise, anti-osteoporosis, osteogenic factor expressions, bone microstructure, bone biomechanics

## Abstract

**Purpose:**

Exercise therapy and key regulators of bone quality exert anti-hyperglycemic effects on type 2 diabetes mellitus (T2DM) mice. A number of programs have been reported to have an effect on bone disease in T2DM. Major unanswered questions concern the potential correlation of exercise with the improvement of bone quality in T2DM mice and how the nonlinear optical properties of bone are correlated with changes to its crystal structure.

**Methods:**

Subjects were randomly divided into six groups: 1) control (C) group, which was fed a normal diet (*n* = 8); 2) T2DM quiet group, which was given a high-fat diet and quiet (*n* = 8); 3) T2DM plus swimming (T2DM+S) group, which received T2DM and swim training (*n* = 8); 4) T2DM plus resistance exercise (T2DM+RE) group, which was given T2DM and resistance exercise (*n* = 8); 5) T2DM plus aerobic exercise (T2DM+AE) group, with T2DM and medium-intensity treadmill exercise (*n* = 8); and 6) T2DM plus high-intensity interval training (T2DM+HIIT), with T2DM and high-intensity variable-speed intervention (*n* = 8). The levels of runt-related transcription factor 2 (*RUNX2*), osterix (*OSX*), and alkaline phosphatase (*ALP*), as well as the bone microstructure and morphometry, were measured at the end of the 8-week exercise intervention.

**Results:**

Compared with the C group, the bone microstructure indexes [bone mineral density (BMD), bone volume/tissue volume (BV/TV), cortical thickness (Ct.Th), and connectivity density (Conn.D)], the bone biomechanical properties (maximum load, fracture load, yield stress, and elastic modulus), and the osteogenic differentiation factors (*RUNX2*, *OSX*, and *BMP2*) of the T2DM group were significantly decreased (all *p* < 0.05). Compared with the T2DM group, there were obvious improvements in the osteogenic differentiation factor (*OSX*) and Th.N, while the separation of trabecular bone (Tb.Sp) decreased in the T2DM+AE and T2DM+HIIT groups (all *p* < 0.05). In addition, the bone microstructure indicators BV/TV, tissue mineral density (TMD), Conn.D, and degree of anisotropy (DA) also increased in the T2DM+HIIT group, but the yield stress and Ct.Th deteriorated compared with the T2DM group (all *p* < 0.05). Compared with the T2DM+S and T2DM+RE groups, the BV/TV, trabecular number (Tb.N), Tb.Sp, and Conn.D in the T2DM+AE and T2DM+HIIT groups were significantly improved, but no significant changes in the above indicators were found between the T2DM+S and T2DM+RE groups (all *p* < 0.05). In addition, the BMD and the expression of *ALP* in the T2DM+AE group were significantly higher than those in the T2DM+HIIT group (all *p* < 0.05).

**Conclusion:**

There was a significant deterioration in femur bone mass, trabecular bone microarchitecture, cortical bone geometry, and bone mechanical strength in diabetic mice. However, such deterioration was obviously attenuated in diabetic mice given aerobic and high-intensity interval training, which would be induced mainly by suppressing the development of T2DM. Regular physical exercise may be an effective strategy for the prevention of not only the development of diabetes but also the deterioration of bone properties in patients with chronic T2DM.

## Introduction

Type 2 diabetes mellitus (T2DM) is a metabolic disease characterized by high blood sugar caused by impaired insulin activity (1). With increasing incidence year by year, T2DM has become a worldwide epidemic. As a serious, long-term condition, T2DM has a negative impact on the life and well-being of individuals, families, and society (2). A number of studies have shown that T2DM is highly significantly associated with an increased risk of hip (3) and ankle and wrist (4) fractures, which is attributed to the change in bone mineral density (BMD) (5, 6). Studies have found that 46.8% of diabetic patients suffer from osteoporosis (OP), which may be due to the dysfunction of cells caused by systemic diseases and the regulation of bone metabolism (7). OP is caused by the degeneration of the bone microstructure, which can lead to an increased risk of fracture and a decrease in bone density and quality (8, 9).

There is still a lot of confusion on the relationship between T2DM and OP. Research shows that a higher body weight and hyperinsulinemia in patients with T2DM may be favorable for enhancing the bone density, which contributes to bone formation (10, 11). However, some studies have shown the negative effects of T2DM on the trabecular bone structure and mechanical properties (12). Researchers have further found that the bone biomechanical properties show considerable impairments in patients with T2DM, which is also a major reason for the increased risk of fracture (13). Although the bone disease caused by T2DM is a common complication (14), there is still a great lack of studies on the cause of the rising risk of fracture in patients with T2DM, which represents a considerably significant and extremely urgent issue.

As is known, physical activity can not only effectively regulate the blood glucose level in T2DM (15, 16) but also play a positive role in increasing BMD, thus is helpful to improving balance (17, 18) and reducing falls (19). Exercise is also effective in preventing chronic diseases (5). Therefore, experts have advised patients with T2DM to have proper physical exercise, which is crucial for the prevention and treatment of diabetes and its complications, including OP (20, 21). In T2DM model rats, 6 weeks of resistance exercise (RE) showed an obvious increase in the bone volume fraction (bone volume/tissue volume, BV/TV) and cortical bone thickness (22). Another study showed that both moderate- and low-intensity treadmill exercise can significantly increase the BMD of the whole bone and distal femur in T2DM model rats (23). Different diseases need appropriate sports type and intensity. However, there is still no systematic study on the effects of exercise on the bone tissue of T2DM model mice. Therefore, the aim of this study was to investigate the effects of exercise on the bone microarchitecture, bone biomechanics, and the osteogenic differentiation genes in mice with T2DM.

## Materials and methods

### Animal

Five-week-old male C57BL/6J mice were purchased from the Model Animal Research Center of Nanjing University (Nanjing, China). The animals were housed in a room with a 12-h light/dark cycle, 21 ± 2°C temperature, and 40%–60% humidity. They were allowed ad libitum access to water and chow. After a week of acclimatization, the mice were randomly divided into two groups: the control diet (C) group (n = 8) and the high-fat diet (HFD) group (n = 40). Mice in the C group were fed a normal diet (Research Diet, D12450J; 10% kcal from fat and 20% kcal from protein, 3.85 kcal/g; SYSE Ltd., Jiangsu, China) for the duration of the experiment, while mice in the HFD group were fed a high-fat diet (Research Diet, D12492; 60% kcal from fat and 20% kcal from protein, 5.24 kcal/g; SYSE Ltd., Jiangsu, China) for 12 weeks.

### Induction of T2DM and exercise groups

**Figure 1:**
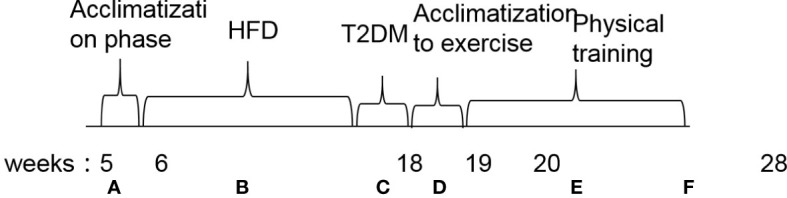
Flowchart of the experimental design. **(A)** Acclimatization phase. **(B)** High-fat diet induction for diabetic animals and commercial feeding in non-diabetic mice. **(C)** Injection of streptozotocin (100 mg/kg) in diabetic groups. **(D)** Acclimatization to exercise. **(E)** Physical training. **(F)** End of experiment. Euthanasia and tissue collection. HFD, high-fat diet; T2DM, type 2 diabetes mellitus.

After 12 weeks, T2DM was induced in mice fed a HFD by a single intraperitoneal injection of streptozotocin (STZ) (Sigma-Aldrich; Merck KGaA, Darmstadt, Germany) dissolved in citrate buffer (pH 4.4) at a dose of 100 mg/kg (24, 25). Control mice received the same volume of citrate buffer. Seven days after STZ injection, fasting blood glucose was measured by blood sampling from the tail vein using a glucometer (Roche Basel, Switzerland). Mice with a fasting blood glucose concentration >13.8 mmol/l were considered diabetic (25 of 30) (25). T2DM mice were randomly divided into four groups: T2DM without exercise (T2DM+NE group, n = 8); T2DM with high-intensity interval training (T2DM+HIIT group, n = 8); T2DM with aerobic exercise (T2DM+AE group, n = 8); T2DM with swim training (T2DM+S group, n = 8); and T2DM with resistance exercise training (T2DM+RE group, n = 8). The experimental technology roadmap is shown in **Figure 1**. All experimental protocols were approved by the Ethics Review Committee for Animal Experimentation of Shanghai University of Sports (approval no. 2016006).

### Body weight measurement

Body weight (BW) was measured at the beginning (5 weeks of age) and at the end of the experiment. Changes in BW were compared among and within the groups pre- and post-modeling.

### Exercise protocol

All mice in the T2DM+HIIT, T2DM+AE, T2DM+S, and T2DM+RE groups were exercised for 60 min/day, 5 days per week, for 8 weeks. The four groups of mice were matched with four different exercise training programs, which were slightly modified from previous reports (26–28). AE involves running on the platform at a speed of 15 m/min without incline, which is a moderate-/low-intensity treadmill exercise (29). In the T2DM+S group, the mice were placed in a 42-cm-long × 40-cm-wide × 36-cm deep container (30), while mice in the T2DM+RE group climbed a 1.1-m vertical ladder with 30% weights attached to their tails. RE was performed three times per week, with three climbs per session and five dynamic movements per climb (26). Group T2DM-HIIT mice underwent 8 weeks of HIIT, the speed of which was 16–26 m/min for 4 min with complete rest for 2 min, in a 10-round cycle, with warm-up and relaxation before and after exercise for 2 min (31). Mice in the T2DM-NE, T2DM-HIIT, T2DM-AE, T2DM-S, and T2DM-RE groups were kept on a HFD during the 8 weeks of exercise (**Figure 1**).

### Tissue collection and handling

After 8 weeks of exercise, the animals were sacrificed by decapitation 48 h after the last training session. The femurs were removed and cleaned of muscles and tendons. Subsequently, the left femurs were washed with saline, frozen in liquid nitrogen, and stored at −80°C for later analysis. The right femurs were immersed in a 4% formaldehyde solution.

### Micro-computed tomography measurements

The influence of T2DM and exercise on the trabecular and cortical bone mass and microstructure was assessed at the right femur metaphysis. The total femur and distal two-thirds of the femur were scanned using VIVACT80 (SCANCO Medical AG, Brüttisellen, Switzerland) at isotropic voxel sizes of 15.6 and 11.0 µm with an X-ray power source of 55 kV, 145 µA, and 8 W.

Three-dimensional (3D) reconstruction and quantitative analyses were performed using SCANCO Medical Evaluation software (SCANCO Medical AG, Brüttisellen, Switzerland). A direct 3D evaluation of the structural parameters of the trabecular and cortical bone was carried out in a region of interest (ROI) that consisted of 110 slices. The start site of the ROI for the trabecular bone was the growth plate, as previously described (26, 32). The cross-sectional geometry of the cortical bone was evaluated at the mid-diaphysis of the tibia, as previously described (26, 32).

The outcomes of the trabecular and cortical microarchitecture included bone volume (BV), tissue volume (TV), BV/TV, bone surface (BS), bone surface/bone volume (BS/BV), BMD, trabecular thickness (Tb.Th), trabecular separation (Tb.Sp), trabecular number (Tb.N), structure model index (SMI), degree of anisotropy (DA), connectivity density (Conn.D), and cortical thickness (Ct.Th).

### Three-point bending test

Biomechanical strength of the left femur was examined using three-point bending (32, 33). Before the test, the bones were thawed at room temperature and then positioned with the posterior condyles downward on a three-point bending machine with a 25-N load cell (TA3200; Leica, Wetzlar, Germany).The femoral bone was placed directly under the testing machine with a span length of 8 mm, which moved at a rate of 1 mm/min until fracture (33). The maximum load (in newton), fracture load (in newton), yield stress (in megapascal), failure strain (*Ɛ*), and elastic modulus (in gigapascal) were calculated using load–displacement curves (34).

### Real-time quantitative PCR

DNAse-treated total RNA was extracted from mouse tibiae using Trizol reagent (DP304; Tiangen Biotech Co., Ltd., Beijing, China). The concentration of the extracted DNA was measured at 260 nm with a microplate reader (BioTek Corporation, Vermont, USA). RNA was converted to complementary DNA (cDNA) using the Takara PrimeScript™ RT reagent Kit (RR037A; Takara, Shiga, Japan). The quantitative PCR (qPCR) reaction system included SYBR Green (Vazyme, Nanjing, China), nuclease-free water, forward and reverse primers (designed and synthesized by Shanghai Shenggong Biology Engineering Technology Service, Ltd.), and DNA, made to a total volume of 20 µl/well. StepOne Plus (Applied Biosystems, Carlsbad, CA, USA) was used for amplification by applying the following parameters: denaturation for 5 min at 95°C, 40 cycles of priming at 95°C for 10 s, and annealing at 60°C for 30 s. The following primers were used: for osterix (*OSX*): 5′-CCTCTTGAGAGGAGACGGGA-3′ (forward) and 5′-TGTACCACGAGCCATAGGGA-3′ (reverse); for runt-related transcription factor 2 (*RUNX2*): 5′-TAGCGTCGTCAGACCGAGA-3′ (forward) and 5′-CAAGGTGCCGGGAGGTAAG-3′ (reverse); for alkaline phosphatase (ALP): 5′-ACTGGCTGTGCTCTCCCTAC-3′ (forward) and 5′-GACCTCTCCCTTGAGTGTGG-3′ (reverse); and for *β-actin*: 5′-CAGCCTTCCTTCTTGGGTATG-3′ (forward) and 5′-AGCTCAGTAACAGTCCGCCT-3′ (reverse). Relative gene expression was calculated and quantified using the 2^−ΔΔCt^ method after normalization to the expression level of *β-actin* RNA.

### Statistical analysis

All data were presented as the mean ± SD. For statistical analysis, SPSS 23.0 software was used. All samples conformed to normal distribution. Comparison of the mean values was performed using an unpaired t-test. Analysis of variance (ANOVA) was utilized when comparing differences between groups, and least significant difference (LSD) was used for post-hoc tests in ANOVA. Statistical difference was considered significant at p < 0.05.

## Results

### HFD/STZ induce changes of the metabolic indexes in C57BL/6 mice

To establish T2DM mice, the animals were administered a HFD and 100 mg/kg STZ injection. It was found that HFD and STZ injection led to increased BW during the induction period (**Table 1**). In addition, compared to mice in the C group, fasting blood glucose was increased in T2DM mice (23.18 ± 0.85 *vs*. 4.80 ± 0.25 mmol/l, *p* < 0.01) (**Table 1**). The above data indicate that the T2DM mouse model was successfully established.

**Table 1:** Animal characteristics.

Characteristics		C	T2DM	*p*-value
Weight (g)	Pre-modeling	21.27 ± 0.97	21.78 ± 1.69	0.535
	Post-modeling	27.90 ± 1.82^##^	41.90 ± 3.16**^##^	<0.001
Fasting blood glucose (mmol/l)		4.80 ± 0.25	23.18 ± 0.85**	<0.001

Data are the adjusted mean ± SD, n = 8 per group.

C, control; T2DM, type 2 diabetes mellitus.

**p < 0.01 vs. C; ^##^p < 0.01 vs. pre-model.

### Osteoporosis was induced in T2DM mice

**Figure 2:**
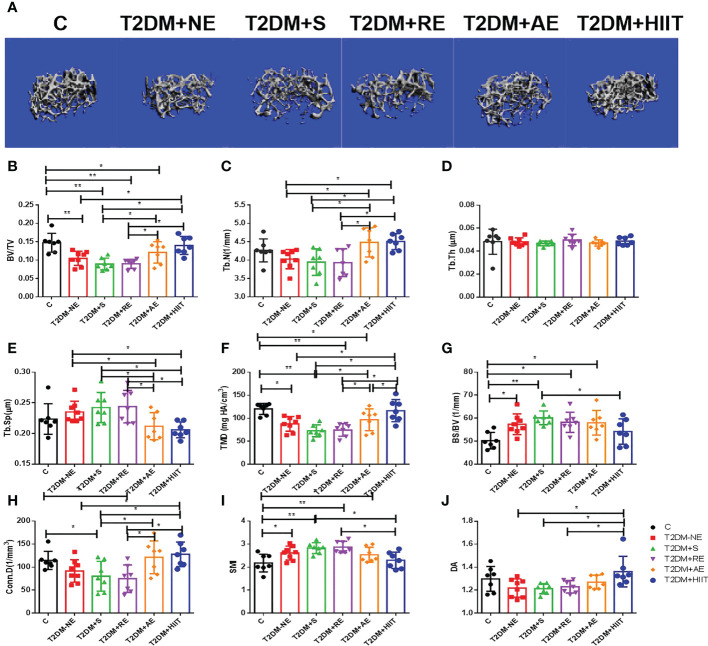
High-fat diet (HFD) decreased the bone volume/tissue volume (BV/TV), tissue mineral density (TMD), and connectivity density (Conn.D) of the trabecular bone in type 2 diabetes mellitus (T2DM) mice. Moreover, HFD increased the bone surface/bone volume (BS/BV) of T2DM mice. High-intensity interval training (HIIT) increased the trabecular number (Tb.N), bone volume/tissue volume (BV/TV), tissue mineral density (TMD), connectivity density (Conn.D), and degree of anisotropy (DA) in T2DM mice compared with the T2DM without exercise (T2DM+NE) group. In addition, the trabecular separation (Tb.Sp) value was reduced in the T2DM+HIIT group. **(A)** Three-dimensional reconstruction plot of the trabecular bone. **(B)** Values of BV/TV. **(C)** Tb.N. **(D)** Trabecular thickness (Tb.Th). **(E)** Tb.Sp values. **(F)** Bone tissue density (TMD). **(G)** Bone surface (BS/BV). **(H)** Conn.D values. **(I)** Structure model index (SMI). **(J)** DA values. Data are the adjusted mean ± SD, n ≥ 7 per group. **p < 0.01, *p < 0.05 [independent-samples t-test was used to analyze the control (C) and diabetes model (T2DM+NE) groups, while one-way ANOVA followed by least significant difference (LSD) post-hoc analysis was applied for comparisons within the physical exercise groups].

**Figure 3:**
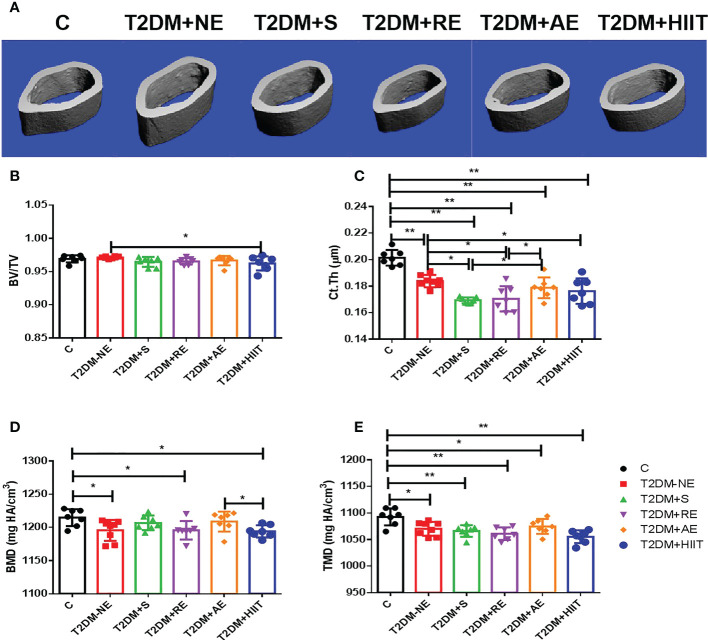
High-fat diet decreased the cortical thickness (Ct.Th) and tissue mineral density (TMD) of the cortical bone in type 2 diabetes mellitus (T2DM) mice. High-intensity interval training (HIIT) increased the bone volume/tissue volume (BV/TV) in T2DM mice. The exercise intervention did not improve the Ct.Th and TMD in T2DM mice. **(A)** Three-dimensional reconstruction plot of the cortical bone. **(B)** Values of BV/TV. **(C)** Ct.Th. **(D)** Bone mineral density (BMD). **(E)** Bone tissue density (TMD). Data are the adjusted mean ± SD, n ≥ 7 per group. **p < 0.01, *p < 0.05 [independent-samples t-test was used to analyze the control (C) and diabetes model (T2DM+NE) groups, while one-way ANOVA followed by least significant difference (LSD) post-hoc analysis was performed for comparisons within the physical exercise groups].

**Figure 4:**
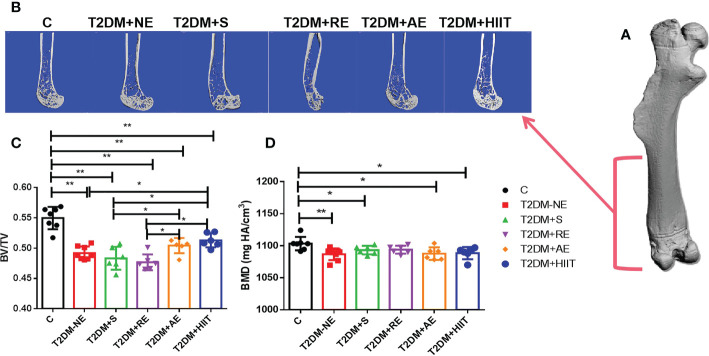
High-fat diet decreased the bone mineral density (BMD) and bone volume/tissue volume (BV/TV) of the whole bone in type 2 diabetes mellitus (T2DM) mice. High-intensity interval training (HIIT) increased the BV/TV in T2DM mice. The BV/TV values in the T2DM+HIIT group were the largest and better than those in the T2DM plus aerobic exercise (T2DM+AE) group when compared to other exercise interventions. **(A)** Three-dimensional (3D) reconstruction plot of the entire femur. **(B)** Reconstruction of the 3D cross-section of the distal femur. **(C)** Values of BV/TV. **(D)** BMD values. Data are the adjusted mean ± SD, n ≥ 7 per group. **p < 0.01, *p < 0.05 [independent-samples t-test was used to analyze the control (C) and diabetes model (T2DM+NE) groups, while one-way ANOVA followed by least significant difference (LSD) post-hoc analysis was conducted for comparisons within the physical exercise groups].

**Figure 5:**
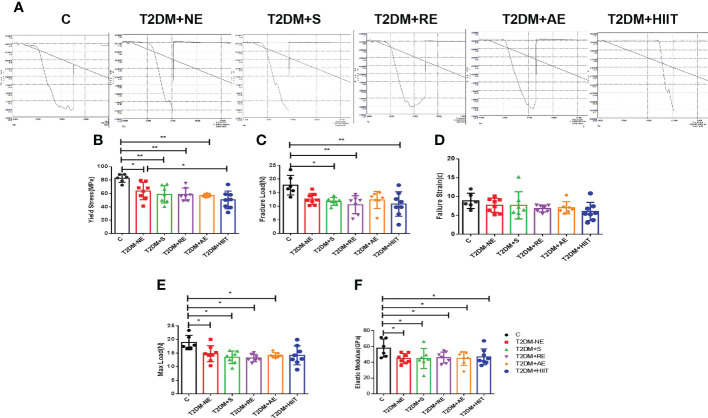
High-fat diet decreased the maximum load, fracture load, yield stress, and elastic modulus in type 2 diabetes mellitus (T2DM) mice. The yield stress of the T2DM plus high-intensity interval training (T2DM+HIIT) group was worse compared with the T2DM without exercise (T2DM+NE) group. **(A)** Mechanical properties measured using three-point bending tests. **(B-F)** Yield stress, maximum load, failure strain, fracture load and elastic modulus. Data are the adjusted means ± SD, n ≥ 6 per group. **p < 0.01, *p < 0.05 [independent-samples t-test was used to analyze the control (C) and diabetes model (T2DM+NE) groups, while one-way ANOVA followed by least significant difference (LSD) post-hoc analysis was performed for comparisons within the physical exercise groups].

**Figure 6:**
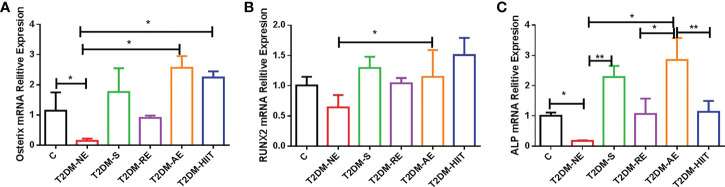
High-fat diet and physical exercise altered the *OSX*, RUNX2, and ALP gene expression in the six groups. RNA from the tibia was isolated, converted to cDNA, quantified by real-time PCR, and normalized to β-actin for *OSX* **(A)**, runt-related transcription factor 2 (RUNX2) **(B)**, and alkaline phosphatase (ALP) **(C)**. The results are presented as the mean fold change in expression ± SD of the control (C) and T2DM groups (n = 3 per group for *OSX*, RUNX2, and ALP; all individual samples were run in triplicate). **p < 0.01, *p < 0.05 [independent-samples t-test was used to analyze the C and diabetes model (T2DM+NE) groups, while one-way ANOVA followed by least significant difference (LSD) post-hoc analysis was employed for comparisons within the physical exercise groups].

The T2DM+NE group showed extensive changes in the morphology of both the trabecular and cortical bone compared to the C group (**Table 2**). There were significant decreases in BV/TV, TMD, and Conn.D of the trabecular bone in the T2DM+NE group compared with the C group (all p < 0.05). Moreover, there was a significant increase in the BS/BV in T2DM mice (p < 0.05) (**Figure 2G**). The cortical bone parameters were also significantly affected, with Ct.Th and TMD reduced in T2DM mice (all p < 0.05) (**Figures 3C, E**). Moreover, the BMD and BV/TV of the whole bone were signaficantly decreased in T2DM mice (all p < 0.05) (**Figures 4B-D**). **Table 3** shows the values of biomechanical parameters of femur in T2DM group and C group. The mechanical parameters (maximum load, fracture load, yield stress, and elastic modulus) were also reduced in T2DM mice (all p < 0.05) (**Figures 5A-F**). HFD caused a decrease in the expression of the *OSX* and ALP genes in all six groups (all p < 0.05) (**Figures 6A, C**).

**Table 2:** Micro-CT analysis of the femur parameters in the control and treatment groups.

	Parameters	C	T2DM+NE	T2DM+S	T2DM+RE	T2DM+AE	T2DM+HIIT
Trabecular bone	Tb.N (1/mm)	4.27 ± 0.31	4.03 ± 0.26	3.95 ± 0.36	3.93 ± 0.38	4.48 ± 0.40	4.51 ± 0.22
	Tb.Th (mm)	0.22 ± 0.25	0.24 ± 0.17	0.24 ± 0.02	0.24 ± 0.03	0.21 ± 0.02	0.21 ± 0.01
	Tb.Sp (mm)	0.05 ± 0.01	0.05 ± 0.003	0.05 ± 0.002	0.12 ± 0.17	0.05 ± 0.002	0.05 ± 0.003
	BS/BV (1/mm)	50.24 ± 3.58	57.35 ± 4.50*	60.15 ± 3.011**	58.21 ± 0.17*	58.00 ± 5.35*	54.19 ± 5.53*^&^
	TMD (mg HA/cm^3^)	120.42 ± 11.92	87.95 ± 15.98*	72.84 ± 13.13**	74.64 ± 13.69**	96.68 ± 23.81*^&$^	116.63 ± 23.88^#&$§^
	BV/TV	0.15 ± 0.03	0.10 ± 0.02**	0.09 ± 0.012**	0.09 ± 0.01**	0.12 ± 0.03*^&$^	0.14 ± 0.02^&$^
	DA	1.30 ± 0.11	1.22 ± 0.08	1.21 ± 0.05	1.23 ± 0.05	1.27 ± 0.06	1.36 ± 0.13^#&$^
	SMI	2.18 ± 0.38	2.61 ± 0.30*	2.83 ± 0.24**	2.88 ± 0.25**	2.54 ± 0.32*	2.30 ± 0.35^&$^
	Conn.D (1/mm^3^)	114.62 ± 19.65	91.74 ± 24.46*	80.29 ± 32.36*	75.08 ± 29.75	121.43 ± 35.67^&$^	121.43 ± 35.66^#&$^
Cortical bone	BV/TV	0.97 ± 0.00	0.97 ± 0.00	0.96 ± 0.00	0.97 ± 0.00	0.96 ± 0.01	0.96 ± 0.01^#^
	Ct.Th (mm)	0.20 ± 0.00	0.18 ± 0.01**	0.17 ± 0.00**^#^	0.17 ± 0.01**#	0.18 ± 0.01**^&§^	0.18 ± 0.01**^#^
	TMD (mg HA/cm^3^)	1,092.60 ± 15.96	1,070.39 ± 113*	1,066.11 ± 11.07**	1,061.04 ± 11.73**	1,074.58 ± 14.06*	1,055.35 ± 12.03**
	BMD (mg HA/cm^3^)	1,214.70 ± 12.76	1,195.51 ± 15.78*	1,206.73 ± 10.97*	1,195.59 ± 14.00*	1,208.70 ± 15.10	1,194.13 ± 9.17^§^
Whole bone	BV/TV	0.55 ± 0.02	0.49 ± 0.01**	0.48 ± 0.02**	0.48 ± 0.01**	0.50 ± 0.01**^&$^	0.51 ± 0.01**^#&$^
	BMD (mg HA/cm^3^)	1,103.45 ± 10.48	1,086.81 ± 9.15**	1,093.25 ± 6.59*	1,093.88 ± 6.19*	1,087.72 ± 9.85*	1,088.53 ± 9.55*

Data are the adjusted mean ± SD, n ≥ 7per group.

Tb.N, trabecular number; Tb.Th, trabecular thickness; Tb.Sp, trabecular separation; BS/BV, bone surface/bone volume; TMD, tissue mineral density; HA, hydroxyapatite; BV/TV, bone volume/tissue volume; DA, degree of anisotropy; SMI, structure model index; Conn.D, connectivity density; Ct.Th, cortical thickness; BMD, bone mineral density; C, control; T2DM, type 2 diabetes mellitus; NE, no exercise; S, swimming; RE, resistance exercise; AE, aerobic exercise; HIIT, high-intensity interval training.

*p < 0.05, **p < 0.01 (vs. the C group); ^#^p < 0.05 (vs. the T2DM+NE group); ^&^p < 0.05 (vs. the T2DM+S group); ^$^p < 0.05 (vs. the T2DM+RE group); ^§^p < 0.05 (vs. the T2DM+AE group).

**Table 3:** Biomechanical parameters obtained from the three-point bending test of the femur.

		C	T2DM+NE	T2DM+S	T2DM+RE	T2DM+AE	T2DM+HIIT
Mechanical	Max load (N)	18.86 ± 2.64	14.76 ± 2.94*(0.038)	13.42 ± 2.33*(0.01)	13.16 ± 1.36*(0.02)	14.18 ± 0.95*(0.018)	14.18 ± 3.50
	Failure strain (*ϵ*)	8.88 ± 2.02	7.69 ± 1.87	7.68 ± 3.62	6.83 ± 0.901	7.05 ± 1.60	6.05 ± 2.41
	Fracture load (N)	17.73 ± 3.57	12.67 ± 2.05	11.76 ± 1.42	10.55 ± 3.29*(0.024)	12.33 ± 3.13	10.81 ± 4.49
	Elastic modulus (GPa)	57.99 ± 11.46	44.85 ± 6.24*0.008)	44.72 ± 12.71*(0.015)	45.93 ± 7.00)*(0.016	44.78 ± 8.60*(0.009)	46.92 ± 10.01*(0.03)
	Yield stress (MPa)	82.70 ± 6.16	63.62 ± 13.01*(0.005)	58.65 ± 12.96**(0.000)	58.48 ± 9.62**(0.001)	57.01 ± 2.44**(0.001)	50.41 ± 13.28*(0.00)

Data are the adjusted mean ± SD, n ≥ 6 per group.

C, control; T2DM, type 2 diabetes mellitus; NE, no exercise; S, swimming; RE, resistance exercise; AE, aerobic exercise; HIIT, high-intensity interval training.

*p<0.05, **p<0.01 (vs. the C group).

### Effect of exercise training on the microstructure of the bone trabeculae in T2DM mice

Compared with the T2DM group, the T2DM+AE and T2DM+HIIT groups showed a significant increase in Th.N, while the Tb.Sp of T2DM mice was significantly decreased (all p < 0.05) (**Figures 2C, E**). In addition, the T2DM+HIIT group also showed increased bone microstructure indicators (BV/TV, TMD, and Conn.D) compared with the T2DM group (all p < 0.05) (**Figures 2B, F, H**). On the other hand, the BV/TV, Tb.N, Tb.Sp, and Conn.D in the T2DM+AE and T2DM+HIIT groups were significantly improved compared with the T2DM+S and T2DM+RE groups, but there was no significant change in the above indicators between the T2DM+S and T2DM+RE groups (all p < 0.05) (**Figures 2B, C, E, F, H**). Similarly, the SMI and DA in the T2DM+HIIT group were significantly improved compared with those in the T2DM+S and T2DM+RE groups (all p < 0.05) (**Figures 2I, J**). In addition, the TMD value in the T2DM+HIIT group was significantly higher than that in the T2DM+AE group (all p < 0.05) (**Figure 2F**). However, there was no significant difference in trabecular thickness among the groups (all p>0.05) (**Figure 2D**).

### Effect of exercise training on the cortical parameters in T2DM mice

BV/TV and Ct.Th decreased after exercise when compared with the T2DM+NE group mice (p < 0.05) (**Figures 3B, C**), but the other cortical bone parameters did not significantly differ after exercise intervention (**Figures 3A-E**). AE significantly increased the Ct.Th (p < 0.05) and BMD (p > 0.05) compared with the other three exercise intervention groups (**Figures 3C, D**).

### Effect of exercise training on the geometry of the whole femur in T2DM mice

As shown in **Figure 4**, the BV/TV of the whole bone was increased in the T2DM+RE, T2DM+AE, and T2DM+HIIT groups compared with the T2DM-NE group (p < 0.05) (**Figure 4B**). However, the BV/TV values in the T2DM+AE and T2DM+HIIT groups were significantly higher than those in the T2DM+S and T2DM+RE groups (p < 0.05) (**Figure 4B**). Additionally, no differences in the BMD among the four exercise interventions were found (p > 0.05) (**Figure 4C**).

### Effect of exercise training on the morphology of the femur in T2DM mice

Compared with the T2DM+NE group, the number of trabeculae increased, the distance of the bone trabeculae decreased, and the arrangement of the bone trabeculae was orderly in the T2DM+HIIT group. Moreover, trabecular dispersion was highest in the T2DM+S group (**Figure 2A**). The bone cortex of the four groups with exercise intervention was generally similar to that of the T2DM+NE group (**Figure 3A**). The longitudinal section also showed that the T2DM+RE group had more severe bone curvature than the other groups (**Figure 4A**).

### Effect of exercise training on the biomechanical index of the femur in T2DM mice

**Table 3** shows the values of biomechanical parameters of femur in each exercise intervention group of T2DM. The T2DM+HIIT group showed worse yield stress compared with the T2DM+NE group (p < 0.05) (**Figure 5B**). The maximum load, fracture load, failure strain, and the elastic modulus were not significantly impacted by training, as shown in **Figures 5C-F** (p < 0.05). These data demonstrate that HIIT reduced the yield stress, but had no other effects on the femur in T2DM mice.

### Effect of exercise training on gene expression in T2DM mice

The T2DM+AE and T2DM+HIIT groups showed an increased relative expression of *OSX* compared with the T2DM+NE group (*p* < 0.01) (**Figure 6A**). The T2DM+AE group also showed increased relative expressions of *RUNX2* and *ALP* compared with the T2DM+NE group (*p* < 0.01) (**Figures 6B, C**). Similarly, the T2DM+S group also showed an increased relative expression of *ALP* compared with the T2DM+NE group (*p* < 0.01) (**Figure 6C**). The relative expression of *ALP* in the T2DM+RE (*p* < 0.05) and T2DM+HIIT (*p* < 0.01) groups was significantly increased compared to that in the T2DM+AE group (*p* < 0.01) (**Figure 6C**).

## Discussion

In this study, we showed that T2DM and exercise had differential effects on the cortical and trabecular bone of the femur when compared to their control counterparts. More specifically, T2DM mice, regardless of exercise status, showed significant negative alterations in the bone microarchitecture indexes (BV/TV, Ct.Th, TMD, and Conn.D) and biomechanical properties (maximum load, fracture load, yield stress, and elastic modulus). On the other hand, the expression levels of the osteogenic differentiation genes (*OSX*, RUNX2, and ALP) were significantly reduced. Negative morphological changes were seen in the total bone and cortical bone of the T2DM mice in this study, which is supported by previous studies (33, 35). Studies comparing the cortical bone and the femur in patients with normal and T2DM osteopathy found that patients with type 2 diabetes had loss of cortical bone mass and significant cortical void (35). This decrease in cortical bone mass and cortical bone volume ratio was also seen in our study and in previous studies (33). Therefore, T2DM model mice injected with STZ after given a HFD could simulate the bone disease of T2DM. The negative changes seen in the trabecular bone of the T2DM mice in this study are supported by previous studies (36, 37). However, our three-point bending data suggest that T2DM induces the deterioration of femoral biomechanical properties, but without significant differences. Our results replicated those of previous reports on mice and rats alike demonstrating changes in the BMD and biomechanical indexes in response to T2DM (5, 6, 38).

It is important to understand the basics of OP prevention behaviors such as adequate calcium intake and regular exercise, which are essential to building and maintaining healthy bones throughout life in individuals with T2DM (39, 40). However, different exercise methods have different effects on the peak bone mass. We found that HIIT and AE, started after skeletal maturity, could reverse some of these negative alterations in the cortical and trabecular bone of T2DM mice, while swimming could exacerbate some of these negative alterations. Additionally, RE caused bone deformation in the femur. Therefore, the exercise interventions in the T2DM group had differential effects on the cortical geometry and trabecular microarchitecture compared to T2DM+NE. However, HIIT significantly reduced the yield stress of the femur (*p* < 0.05), and the failure load capacity was also the worst (*p* > 0.05). In addition, AE also showed increased relative expressions of *OSX*, *RUNX2*, and *ALP* compared with T2DM+NE.

Previous studies have shown that exercise therapy can effectively prevent bone loss in patients with OP (41, 42). Therefore, we analyzed the effects of four types of exercise interventions on the bone microstructure and bone biomechanics of T2DM mice and determined the most suitable exercise for alleviating OP. Swimming can improve cardiopulmonary function (43), reduce the level of blood lipid (44), improve the collective antioxidant capacity, and delay aging (45). Previous studies have shown that swimming leads to lower bone mass in teenagers or college students (46–48). On the contrary, other studies have also shown that swimming can increase bone mass in postmenopausal women (49–51), which might explain the promotion of osteoclast-led bone resorption by swimming exercise (49). These observations demonstrate that swimming reduces cortical bone volume and thickness and, therefore, is not suitable for improving the bone microstructure and biomechanical properties of patients with T2DM. These results are consistent with previous studies, which may have been due to the lack of gravity activation (52). However, in this study, swim training significantly increased the expression of *ALP* in the T2DM+S group. Swimming also caused a significant increase in BW and in *RUNX2* mRNA expression, while the trabecular morphological structure of the distal femur and the indexes of bone histomorphology were not significantly improved in T2DM mice (30). Swim training significantly increased the *RANKL*/*OPG* ratio compared to the diabetic group (53, 54). Based on analysis of the literature and the results of this experiment, swim training may promote the expression of early osteogenic differentiation factors, but the reduction of gravity stimulation will significantly promote bone absorption, which will lead to the reduction of bone mass.

A lot of systematic reviews and meta-analyses have shown an increase in the quality of life (55) and the physical function and body composition (56) in patients when practicing RE. Previous studies have shown that RE is beneficial for bone mass (57–60). Some recent studies have reported that RE combined with medication increased the BMD, but RE alone did not affect BMD (61, 62). Our micro-CT (μCT) data on the cortical bone also suggest that RE did not affect the measures of the microstructure and bone biomechanics, except that it reduced the Ct.Th in T2DM mice. The RE group showed poor performance in terms of bone microstructure and bone biomechanics of the trabecular bone in the four T2DM groups given exercise training. These observations demonstrated that RE reduced the Ct.Th and caused bone deformation in T2DM. Therefore, RE is not suitable for improving the bone microstructure and biomechanical properties of patients with T2DM. However, these results indicate that the 6-week resistance training regimen effectively increased the BMD and improved bone quality in the proximal tibial metaphyseal trabecular bone of T2DM model rats (22). It is possible that RE exerts different effects on the bone quality of the tibia and femur.

Previous studies have shown that moderate-/low-intensity treadmill exercise could increase femoral BMD in T2DM mice (63–65). In addition, previous μCT scans and mechanical tests revealed that the trabecular bone microarchitecture and bone mechanical properties of T2DM mice were improved after 8 weeks of treadmill exercises (66). Our μCT data also suggested that the AE group had better performance in terms of bone microstructure and bone biomechanics. However, the bone biomechanics of the T2DM+AE group was not significant compared to the T2DM+NE group. The duration of treadmill exercise in our study was only 8 weeks, which may have caused AE improvement, but with no significant difference. As reported by Minematsu et al., wheel-running for 17 months could prevent the deterioration of the bone properties (tibia bone mass and trabecular bone microarchitecture) in T2DM rats (67). Furthermore, the combination of teriparatide and treadmill exercise increased the BMD and trabecular and cortical bone strength of the femur with improved microarchitecture in T2DM model rats (68). Thus, a longer duration of AE and diet could induce significant improvements in the bone microstructure and bone biomechanics of T2DM mice.

Our μCT data suggested that the HIIT group had the best performance in terms of bone microstructure in the femur bone in the four T2DM groups given exercise training. Our results are in accordance with previous studies showing that HIIT with resistance training can significantly increase the BMD (69, 70). Eight weeks of HIIT and downhill running exercise mediated the Meg3/P62/Runx2 pathway to promote bone formation in T2DM mice (31). However, except for *OSX*, HIIT did not significantly improve the expression of *RUNX2* and *ALP* compared with the T2DM+NE group. In addition, studies have shown that a 10- or 12-week HIIT intervention did not induce significant changes in BMD (71, 72). The differences in the results of this experiment could be due to the short duration of the intervention program or the intervention program without resistance training since longer programs and resistance training are needed to induce improvements in BMD (49). These data demonstrate that AE and HIIT are suitable exercises for the alleviation of OP in patients with T2DM compared to the other three types of exercise.

The present study has some limitations. Firstly, this study did not track the weight, blood sugar, and body composition of each exercise intervention group. Secondly, the molecular mechanisms of T2DM and exercise intervention on bone disease were not further explored. Finally, different exercise intensities, exercise cycles, and other factors will lead to different experimental results. Therefore, more detailed exercise programs need to be designed in order to verify our findings.

Significant deterioration was observed in femur bone mass, trabecular bone microarchitecture, cortical bone geometry, and bone mechanical strength in diabetic mice. However, such deterioration was obviously attenuated in diabetic mice given AE and HIIT, which would be induced mainly by suppressing the development of T2DM. Regular physical exercise may be an effective strategy for the prevention of not only the development of diabetes but also the deterioration of bone properties in patients with chronic T2DM.

## Data availability statement

The original contributions presented in the study are included in the article/supplementary material. Further inquiries can be directed to the corresponding authors.

## Ethics statement

The animal study was reviewed and approved by Shanghai University of Sports (approval no. 2016006). Written informed consent was obtained from the owners for the participation of their animals in this study.

## Author contributions

MZ and YL performed the experiment. MZ wrote the manuscript. MZ, LL, MH, MW, and JZ reviewed the manuscript. All authors contributed to the article and approved the submitted version.

## Funding

Funding support for this work was from Shanghai Frontiers Science Research Base of Exercise and Metabolic Health, the Research Program of Exercise and Public Health (0831) in Shanghai University of Sport. This work was also supported by Shanghai Key Laboratory for Human Athletic Ability Development and Support (Shanghai University of Sport) (no. 11DZ2261100).

## Conflict of interest

The authors declare that the research was conducted in the absence of any commercial or financial relationships that could be construed as a potential conflict of interest.

## Publisher’s note

All claims expressed in this article are solely those of the authors and do not necessarily represent those of their affiliated organizations, or those of the publisher, the editors and the reviewers. Any product that may be evaluated in this article, or claim that may be made by its manufacturer, is not guaranteed or endorsed by the publisher.
